# Isolation, genomic characterization, and safety assessment of an *O*-desmethylangolensin-producing *Clostridium beijerinckii* strain from Chinese Stinky Tofu

**DOI:** 10.1128/spectrum.00632-26

**Published:** 2026-09-17

**Authors:** Xia Guo, Sifan Zhang, Ziyi Cui, Ying Zhang, Zixuan Zhao, Guanmian Wei, Hao Li, Xiu-Ling Wang

**Affiliations:** 1 College of Life Sciences, Hebei Agricultural University 162640, Baoding, Hebei, China; 2 College of Food Science and Technology, Hebei Agricultural University 74562, Baoding, Hebei, China; University of Melbourne, Melbourne, Australia

**Keywords:** safety assessment, genomic characterization, *O*-desmethylangolensin, isoflavone biotransformation, isolation

## Abstract

The health benefits of dietary soy isoflavones are largely mediated by specific microbial metabolites, such as *O*-desmethylangolensin (*O*-DMA). However, the diversity and application potential of *O*-DMA-producing strains remain poorly explored, primarily due to the limited availability of isolated strains, narrow ecological sources, and a lack of practical applications. In this study, an *O*-DMA-producing bacterium, designated strain FRJF5, was isolated from Chinese stinky tofu under anaerobic conditions and was identified as *Clostridium beijerinckii*. The biosynthesized *O*-DMA exhibited an enantiomeric excess (e.e.) of 78.6%. Based on phylogenetic and average nucleotide identity analyses against 235 public *C. beijerinckii* genomes, the clustering of FRJF5 with strains from diverse habitats—including industrial fermentation settings, animal feces, and soil—highlights the broad ecological diversity within this species. Functional gene mining and intra-species comparative genomics revealed a unique flavonoid metabolism gene cluster in FRJF5. Using apigenin as a representative flavonoid, we confirmed the successful conversion to 3-(4-hydroxyphenyl)-propionic acid. Moreover, the strain was predicted and verified to possess a substantial butyrate-producing capacity. Genomic screening for virulence or antibiotic resistance genes, combined with phenotypic tests (hemolysis, antibiotic susceptibility, and mouse gavage), revealed a favorable safety profile for strain FRJF5. Finally, intervention experiments in a mouse model of colitis supported its potential in alleviating the disease. Collectively, this study identifies *C. beijerinckii* FRJF5 as a strain capable of simultaneously producing *O*-DMA and butyrate, highlighting its potential for future applications in functional foods.

**IMPORTANCE:**

Soy isoflavones require gut bacterial conversion into bioactive metabolites—such as the anti-inflammatory compound *O*-desmethylangolensin (*O*-DMA)—to exert health benefits. Yet *O*-DMA-producing strains remain scarce, largely confined to fecal sources, and poorly characterized. Here, we isolated *Clostridium beijerinckii* FRJF5 from Chinese stinky tofu, an unexplored ecological niche. This strain not only produces enantiomerically enriched *O*-DMA but also co-produces butyrate, a metabolite known to strengthen gut barrier function. Genomic mining uncovered a unique flavonoid metabolism gene cluster responsible for this dual activity. Combined with favorable safety profiles, FRJF5 emerges as a strong candidate for functional food applications. This work expands the known diversity of *O*-DMA producers and bridges traditional fermented foods with next-generation probiotic development.

## INTRODUCTION

In addition to being rich in high-quality protein, soybeans also contain functional components such as isoflavones, making them a health food for humans. Soy isoflavones are a class of secondary metabolites produced during the growth of soybeans and a few other legumes. In 1931, Walz et al. first isolated soy isoflavones from soybeans (1). It is now known that daidzein and genistein, along with their respective glycosides, are the primary components of soy isoflavones (2). Humans primarily obtain soy isoflavones from foods such as soybeans (3). Soy isoflavones share a chemical structure similar to mammalian estrogen, allowing them to bind to estrogen receptors and exert estrogen-like effects (4). Numerous epidemiological studies have shown that the incidence of breast and prostate cancers is significantly lower among Eastern populations compared to Western populations. One contributing factor to this finding is the high level of isoflavone consumption due to frequent consumption of soy products. Beyond their anticancer activity, soy isoflavones also offer various physiological benefits, such as reducing the risk of cardiovascular and cerebrovascular diseases and preventing osteoporosis (5–7).

In the 1990s, research first confirmed that the human gut microbiota can convert ingested soy isoflavones into various metabolites, including dihydrodaidzein (DHD), dihydrogenistein (DHG), equol, 5-hydroxy-equol, and *O*-desmethylangolensin (*O*-DMA) (8, 9). Since 2000, a series of bacterial strains from the gut microbiota of different animals that exhibit diverse biotransformation capabilities for soy isoflavones have been isolated by researchers, and more biological activities of microbially derived soy isoflavone metabolites have been reported. The results of both others’ and our own research have shown that soy isoflavone metabolites, including those mentioned above, are more biologically active than their precursors daidzein and genistein (10–12). Numerous bacterial strains with soy isoflavone biotransforming activity, most of which are obligate anaerobes, have been isolated and identified. For a long time, it has been widely believed that natural isoflavones present in soybeans or soy-based foods can only be metabolized into different products after being ingested by humans or other animals. Therefore, samples used for isolating and screening bacteria that transform soy isoflavone have typically been fresh feces from humans or other animals. In 2013, Abiru et al. first isolated an obligately anaerobic bacterium belonging to the family Coriobacteriaceae from stinky tofu brine, which could convert the soy isoflavone daidzein into *S*-equol. This finding fundamentally challenged the traditional assumption that soy isoflavone-transforming bacteria could only originate from gut microbiota (13). In 2022, Landete et al. heterologously expressed the equol biosynthesis gene cluster in *Limosilactobacillus fermentum*, producing a soy beverage containing *O*-DMA and 6-hydroxy-*O*-desmethylangolesin through fermentation using the engineered strain *L. fermentum* INIA P584L (14). Also in 2022, we isolated for the first time a facultatively anaerobic strain of *Lactobacillus acidipiscis* from stinky tofu, capable of reducing the soy isoflavones daidzein and genistein into DHD and DHG, respectively. Using this strain to ferment soymilk, we successfully produced fermented soy milk containing the isoflavone reduction metabolites DHD and DHG (15).

Through this study, we have for the first time isolated an endospore-forming strictly anaerobic bacterium, *Clostridium beijerinckii* FRJF5, from the traditional Chinese fermented food stinky tofu. This strain is capable of converting daidzein into *O*-DMA and exhibits butyrate-producing ability. Although *Clostridium beijerinckii* exerts potential beneficial effects on host health (16, 17); previous studies have reported that its closely related species such as *Clostridioides difficile* act as critical opportunistic pathogens (18). Meanwhile, *Clostridium butyricum* displays both advantageous (19) and adverse (20) properties relevant to host health. Therefore, the safety of our isolated strain FRJF5 requires comprehensive evaluation. We sequenced the genome of the isolated *C. beijerinckii* FRJF5. Through comparative genomic analysis, we characterized the distribution of antibiotic resistance genes (ARGs) and virulence factors in *C. beijerinckii* FRJF5, and we further assessed its hemolytic activity and antibiotic resistance through experimental tests. Additionally, to evaluate the *in vivo* toxicity of the strain, we conducted safety assessments in mice and intervention studies in mouse models of colitis. This work aims to promote the application of the food-derived *O*-DMA-producing strain *C. beijerinckii* FRJF5 in the development of functional foods.

## MATERIALS AND METHODS

### Media and chemicals

The culture broths used in this study were as follows. Brain heart infusion (BHI) broth was from Difco Co. (Sparks, MD, USA), and reinforced clostridial medium (RCM) was from Guangdong Zhongshan Baiwei Biotechnology Co., Ltd. (Guangdong, China). The following reagents were used as standard substances: daidzein from Indofine (Somerville, NJ, USA), apigenin, naringenin, 3-(4-hydroxyphenyl)-propionic acid from Shanghai Yuanye Bio-Technology Co., Ltd. (Shanghai, China), and *O*-DMA (biosynthesized in our laboratory) (21).

### Bacterial isolation and identification

A sealed glass jar containing fermented bean curd was transferred into an anaerobic chamber (Concept 400; Ruskinn, Mid Glamorgan, UK). Using sterile forceps, a cube of fermented bean curd was taken and cut with a pre-sterilized surgical scalpel; a piece measuring 0.5 cm × 0.5 cm × 0.5 cm was excised from the interior and placed into 1 mL of fresh BHI medium. The mixture was vortexed to disperse the fragments, followed by a pre-incubation period of 18 h in an anaerobic chamber containing 5% CO_2_, 10% H_2_, and 85% N_2_ at 37°C. Subsequently, serial dilutions were prepared and spread onto agar plates. Single colonies were picked and inoculated into 1 mL of fresh BHI medium supplemented with daidzein at a final concentration of 0.1 mmol∙L^−1^, then incubated in the anaerobic workstation for 3 days.

The 16S rRNA gene sequence of the positive strains was amplified with universal primer 27F (5′-AGAGTTTGATCCTGGCTCAG-3′)−1492R (5′-GGTTACCTTGTTACGACTT-3′). The taxonomy of the 16S rRNA gene was aligned against the EzBiocloud database using Blastn. A phylogenetic tree was constructed through the maximum likelihood method using MEGA v7.0. Strain FRJF5 has been deposited in the China General Microbiological Culture Collection Center (CGMCC) under deposit number CGMCC 7.759. The complete genome sequence of isolate FRJF5 was obtained using Illumina and PacBio sequencing platforms. Its whole-genome sequence has been deposited in GenBank under accession no. JCAMPI000000000 and BioProject no. PRJNA1481890.

### Identification of the metabolites of isoflavones and flavonoids

Separated colonies were cultured in medium supplemented with substrate. After 48 h of incubation at 37°C, samples were taken for high-performance liquid chromatography (HPLC) analysis. The substrate and transforming products were extracted as previously described (22), and isoflavones were determined by previous methods. The flavonoids were determined with minor modification by HPLC (Waters 2695 HPLC system) using a Kromasil-C18 column (250 mm × 4.6 mm, 5 μm) and a UV diode array detector (Waters 2996). For the analysis of the enantiomers of the metabolite *O*-DMA, we used previously reported methods (21).

### The safety phenotypic analysis of the strain *C. beijerinckii* FRJF5

The hemolytic activity of *C. beijerinckii* FRJF5 was assessed on Columbia blood agar plates. The strain was incubated at 37°C for 24 h, followed by the measurement of α, β, or γ hemolysis. Blank-control and *Staphylococcus aureus* ATCC 27217 control groups were set up simultaneously.

The antibiotic sensitivity of *C. beijerinckii* FRJF5 to eight common antibiotics belonging to eight categories (penicillins, fluoroquinolones, phenylpropanol, cephalosporins, carbapenems, tetracyclines, linkamides, and nitroimidazoles) was determined by the anaerobic agar dilution method according to the Clinical and Laboratory Standards Institute (CLSI) guidelines (23) issued in 2019. The minimum inhibitory concentration (MIC) was determined according to the CLSI standard.

### Acquisition and annotation of public genomic data sets

A total of 11 complete genomes and 224 MAGs of *C. beijerinckii* were obtained from NCBI RefSeq database. The quality of the genomes, including strain FRJF5, was assessed using CheckM2. Genomes meeting the criteria of >95% completeness and <5% contamination were subsequently annotated for taxonomy with GTDB r214. Gene prediction was performed with Prokka (24) to standardize the input data.

Flavonoid transformation genes were identified via BLASTp, using as queries flavone reductase (FLR) (ADK16070.1), flavanone/flavanonol-cleaving reductase (FCR) (ADK16884.1), and phloretin hydrolase (PHY) (ADK16072.1) from *Clostridium ljungdahlii* DSM 13528, along with CHI (ANU42126.2) from *Flavonifractor plautii* ATCC 49531. Functional annotation was performed against the CAZy database using default parameters. Additionally, butyrate synthesis genes were identified based on EC numbers and BLASTp analysis. Specialized functional databases or software were used to annotate drug resistance genes (DeepARG software) (25), virulence factors of bacterial pathogens (VFDB database), pathogen-host interactions (PHI database) (26), and insertion sequences (ISs) based on the ISfinder database (27). Additionally, we used PHASTEST (28) and IslandViewer 4 server (accessed in March 2025) (29) to identify prophages and predict genomic islands (GIs), respectively, for the strain FRJF5.

### Comparative genomics of *C. beijerinckii*

The average nucleotide identity (ANI) was calculated using fastANI (30) to analyze and evaluate the chromosome identity. In addition, we performed a pangenome analysis using the Roary pipeline (31). We performed phylogenetic analysis at the strain level for the genome, employing the default core marker gene database of PhyloPhlAn (32).

### Animals and experimental design

Seven-week-old specific pathogen-free (SPF) C57BL/6J mice were obtained from Beijing Vital River Laboratory Animal Technology Co., Ltd. All mice were acclimated to the laboratory environment, after which the temperature (23°C ± 2°C), humidity (50% ± 5%), and light-dark cycle were maintained. After 1 week of adaptation, mice were subsequently advanced to the formal experimental protocol. In a 14-day short-term repeated-dose toxicity study, mice were administered the FRJF5 bacterial strain via oral gavage (200 μL∙d^−1^; 1 × 10^9^ CFU∙mL^−1^) (33). Subsequently, male mice were randomly divided into three groups (six mice per group): the normal control group (NC), DSS group (DSS), and *C. beijerinckii* FRJF5 gavage group (Cb). The mice in the Cb group were gavaged with *C. beijerinckii* FRJF5 (200 μL∙day^−1^; 1 × 10^9^ CFU∙mL^−1^), and 200 μL∙day^−1^ PBS was administered to the mice in the NC and DSS groups for 14 consecutive days. During the 8th–14th day period, the sterile drinking water of the DSS and Cb groups was replaced with 2.5% (wt/vol) DSS (P Bio, Canada) to induce colitis. Mice were individually housed, and at a fixed time each day, the amount of food supplied (*W*_0_) was weighed. After 24 h, remaining chow (*W*_1_) and spilled chow (*W*_2_) were collected and weighed. Daily food intake was calculated as *W*_0_‒*W*_1_‒*W*_2_ and expressed as g/cage/day. Results are presented as mean ± SD.

### Assessment of disease activity and pathological changes

A disease activity index (DAI) score was recorded daily after the initiation of DSS administration to further quantify the colitis severity. The DAI score (0–12) consists of three parts based on the clinical features: weight loss, fecal blood, and consistency (34). Short-chain fatty acids (SCFAs) in colon content were measured using Waters 2565/2996 HPLC systems with minor modification (35).

### 16S rRNA gene amplicon sequencing

Each fecal sample was amplified, and the resulting products were subsequently pooled in equimolar amounts. The pooled PCR product was sequenced using the PacBio Revio platform with single-molecule real-time (SMRT) sequencing. Raw sequencing reads were demultiplexed based on barcode and primer sequences, and quality-filtered using Seqkit (v2.9.0). Operational taxonomic units (OTUs) were clustered at 97% identity using the UPARSE algorithm (v7.0.1001). Taxonomic assignment of OTUs was performed using the Mothur algorithm against the SILVA 138.1 database.

## RESULTS

### Isolation and identification of daidzein-biotransforming bacteria

**Fig 1:**
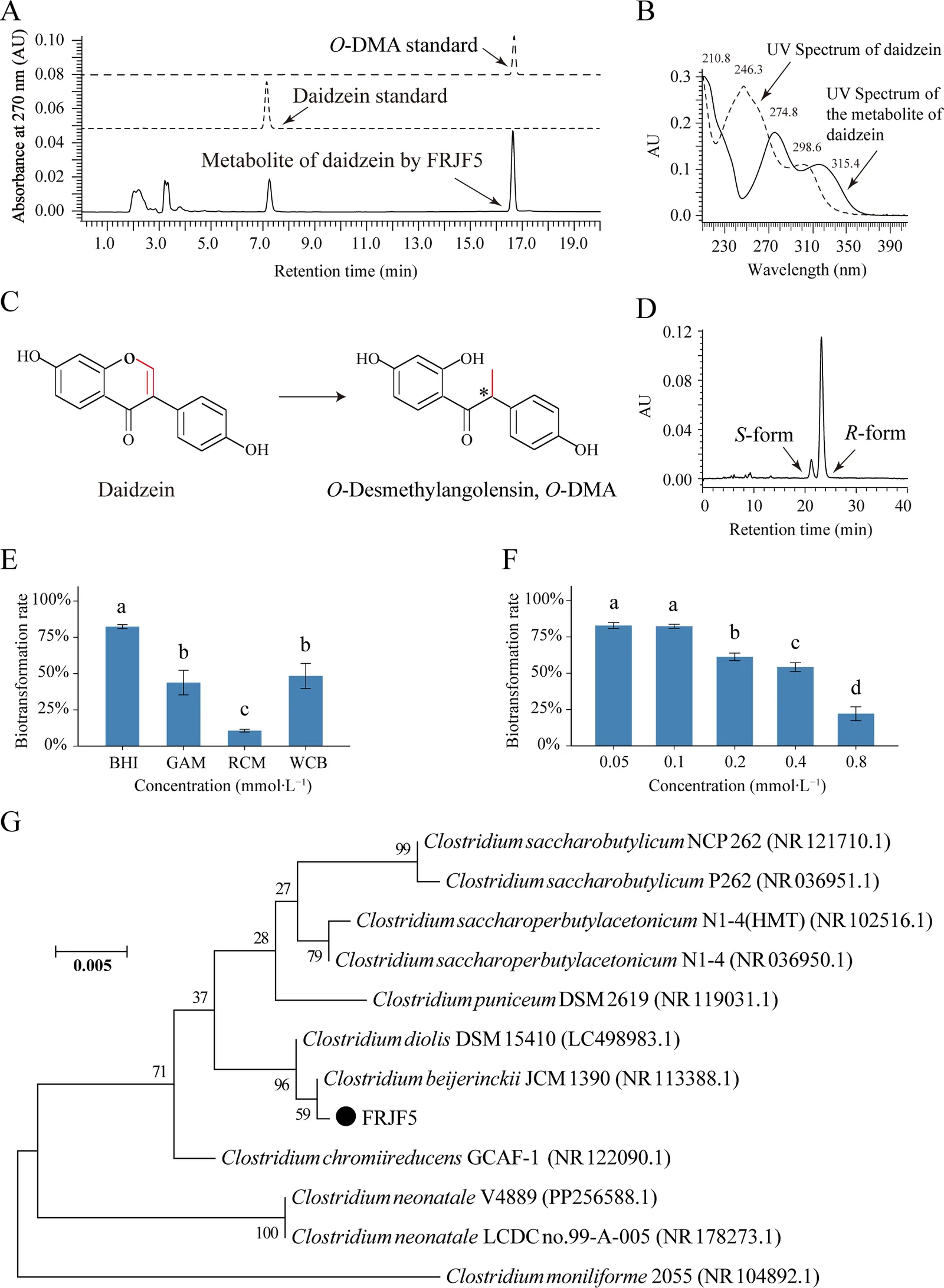
Metabolic characteristics and taxonomic identification of *C. beijerinckii* FRJF5. (**A**) HPLC detection of the conversion of daidzein to *O*-DMA by *C. beijerinckii* FRJF5. (**B**) UV spectra of daidzein and the corresponding metabolite. (**C**) The C-ring cleavage pathway of daidzein that produces *O*-DMA by strain FRJF5. (**D**) Chiral chromatogram of *O*-DMA converted by strain FRJF5. (**E**) Conversion efficiency to 0.1 mmol∙L^−1^ daidzein in different media. (**F**) The bioconversion rate of the strain to different concentrations of daidzein. (**G**) Maximum likelihood phylogenetic tree of strain FRJF5 based on its 16S rRNA gene sequence. Data are presented as mean ± SD (*n* = 3). Different letters indicate significant differences determined by one-way ANOVA followed by Waller-Duncan post hoc test (*P* < 0.05), while identical letters indicate no statistically significant differences (*P* ≥ 0.05).

A strictly anaerobic bacterial strain with the ability to transform soy isoflavones was isolated from stinky tofu and designated as strain FRJF5. HPLC analysis revealed that the retention time of the substrate daidzein was 7.4 min. After biotransformation by strain FRJF5, a new peak appeared at a retention time of 16.7 min, which exactly matched the retention time of the *O*-DMA standard. Furthermore, this new peak exhibited maximum UV absorption at 278 and 317 nm, consistent with the reported UV absorption profile of *O*-DMA (21) (Fig. 1A and B). Additionally, mass spectrometry analysis (ESI-MS) showed that the metabolite derived from daidzein transformation by strain FRJF5 had an [M-H]^−^ ion at *m*/*z* 257, indicating a molecular weight of 258. This aligns with the molecular weight of *O*-DMA (C_15_H_14_O_4_). Therefore, based on HPLC retention time, UV absorption spectrum, and mass spectrometry data, the metabolite produced from daidzein by strain FRJF5 was conclusively identified as *O*-DMA (Fig. 1C). Since *O*-DMA contains a chiral carbon atom, chiral-phase HPLC analysis was performed on the *O*-DMA produced by strain FRJF5 from daidzein. Two peaks were observed at retention times of 20.7 and 22.6 min (Fig. 1D). According to previous reports (36, 37), the earlier peak was identified as the *S* form of *O*-DMA and the later peak as the *R* form. The enantiomeric excess (e.e.) of the produced *O*-DMA was calculated to be 78.6%.

Furthermore, it was observed that different culture media significantly influenced the conversion of daidzein by strain FRJF5. The highest biotransformation activity was observed in BHI medium, followed by Wilkins-Chalgren broth (WCB) and Gifu anaerobic medium (GAM), while the weakest activity occurred in reinforced clostridial medium (RCM) (Fig. 1E). To further investigate the substrate conversion capacity of strain FRJF5 in BHI medium, the strain was co-cultured with varying concentrations of daidzein. The results show that when the daidzein concentration varied from 0.05 to 0.10 mmol∙L^−1^, strain FRJF5 exhibited high conversion efficiency, with average conversion rates of 82.88% and 82.36%, respectively. However, when the substrate concentration increased to 0.20 and 0.40 mmol∙L^−1^, the conversion capacity decreased notably, with average conversion rates of 61.30% and 54.23%, respectively. When the substrate concentration further rose to 0.8 mmol∙L^−1^, the average rate of daidzein conversion by strain FRJF5 dropped to 22.13% (Fig. 1F). Finally, 16S rRNA gene sequencing of strain FRJF5 was performed. Sequence alignment revealed that strain FRJF5 shared the highest identity (99.86%) to *C. beijerinckii* JCM1390. Based on the phylogenetic analysis of strain FRJF5, it was identified as *C. beijerinckii* and designated as *C. beijerinckii* FRJF5 (accession no. PX765463; Fig. 1G).

### Genome analysis of *C. beijerinckii* FRJF5

**Fig 2:**
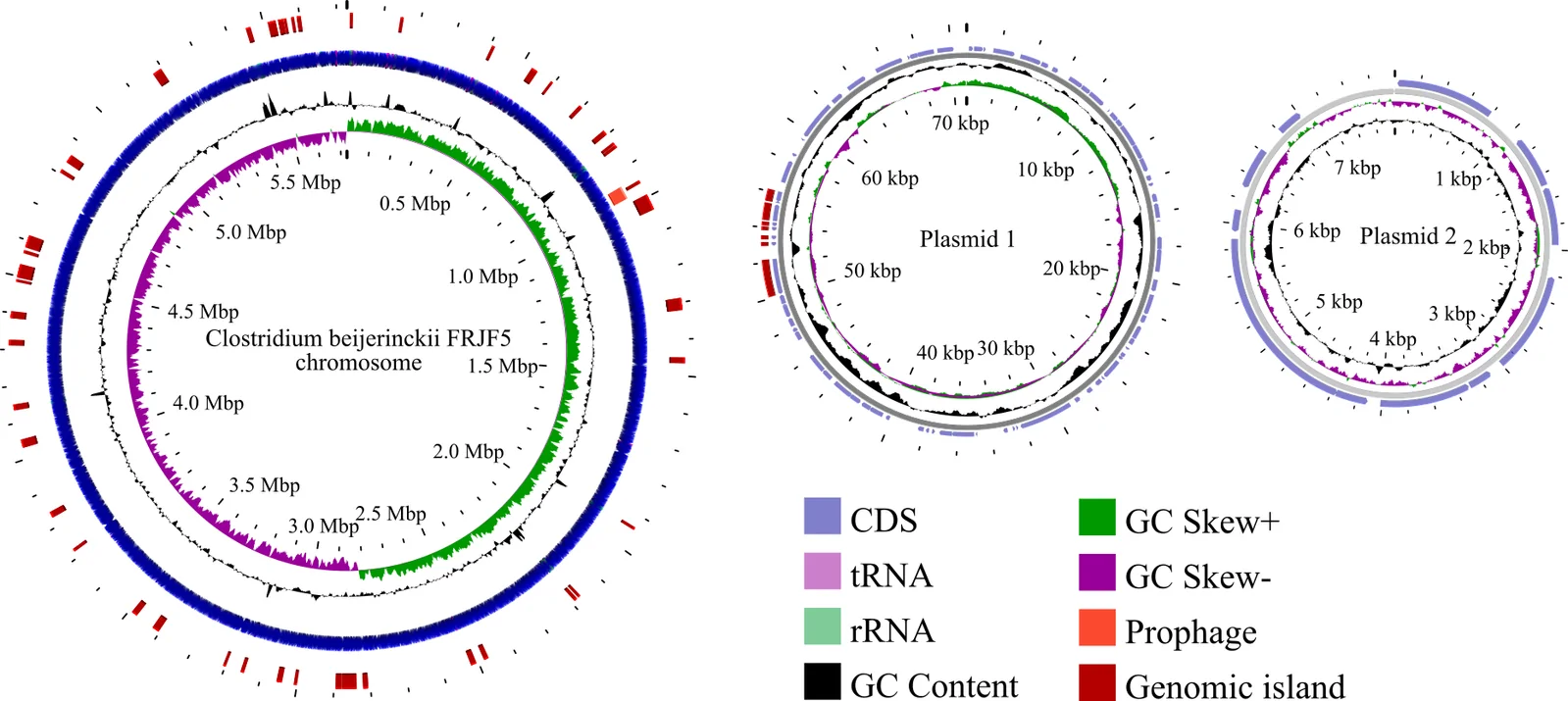
Circular genome mapping of *C. beijerinckii* FRJF5.

The complete genome of *C. beijerinckii* FRJF5 consists of a single chromosome of 5,728,266 bp (29.84% GC content), one non-circular plasmid (plasmid 1, 70,925 bp, 28.86% GC content), and one circular plasmid (plasmid 2, 7,793 bp, 27.52% GC content). The *C. beijerinckii* general genomic features of strain FRJF5 are summarized in Table 1. A total of 5,351 protein-coding sequences (CDS) were predicted, of which 5,261 are located on the chromosome, and 78 and 12 are found on plasmid 1 and plasmid 2, respectively. Multiple GIs were predicted to be scattered across the chromosome of FRJF5, along with one suspect prophage region spanning 38.5 kb and containing several transposases. Additionally, one GI region was predicted on the larger plasmid (Fig. 2). These genomic features suggest that the genome of FRJF5 exhibits considerable plasticity and has a higher potential for genetic exchange with other species.

**TABLE 1:** MIC (µg·mL^−1^) interpretation of *C. beijerinckii* FRJF5 detected by per CLSI guidelines

Category of drug	Antimicrobial	MIC value (interpretation^*a*^)
Penicillins	Ampicillin	0.064 (S)
Tetracyclines	Tetracycline	0.125 (S)
Lincosamides	Clindamycin	0.5 (S)
Cephalosporins	Cefoxitin sodium	4 (S)
Carbapenems	Doripenem	0.5 (S)
Fluoroquinolones	Moxifloxacin	0.5 (S)
Phenicols	Chloramphenicol	4 (S)
Nitroimidazoles	Metronidazole	0.5 (S)

^*a*^ Interpretation of MIC values S (susceptible), I (intermediate), and R (resistant) according to the CLSI﻿.

### Comparative genome analysis of *C. beijerinckii* strains

#### Genomic comparison and pan-genome analysis of *C. beijerinckii*

**Fig 3:**
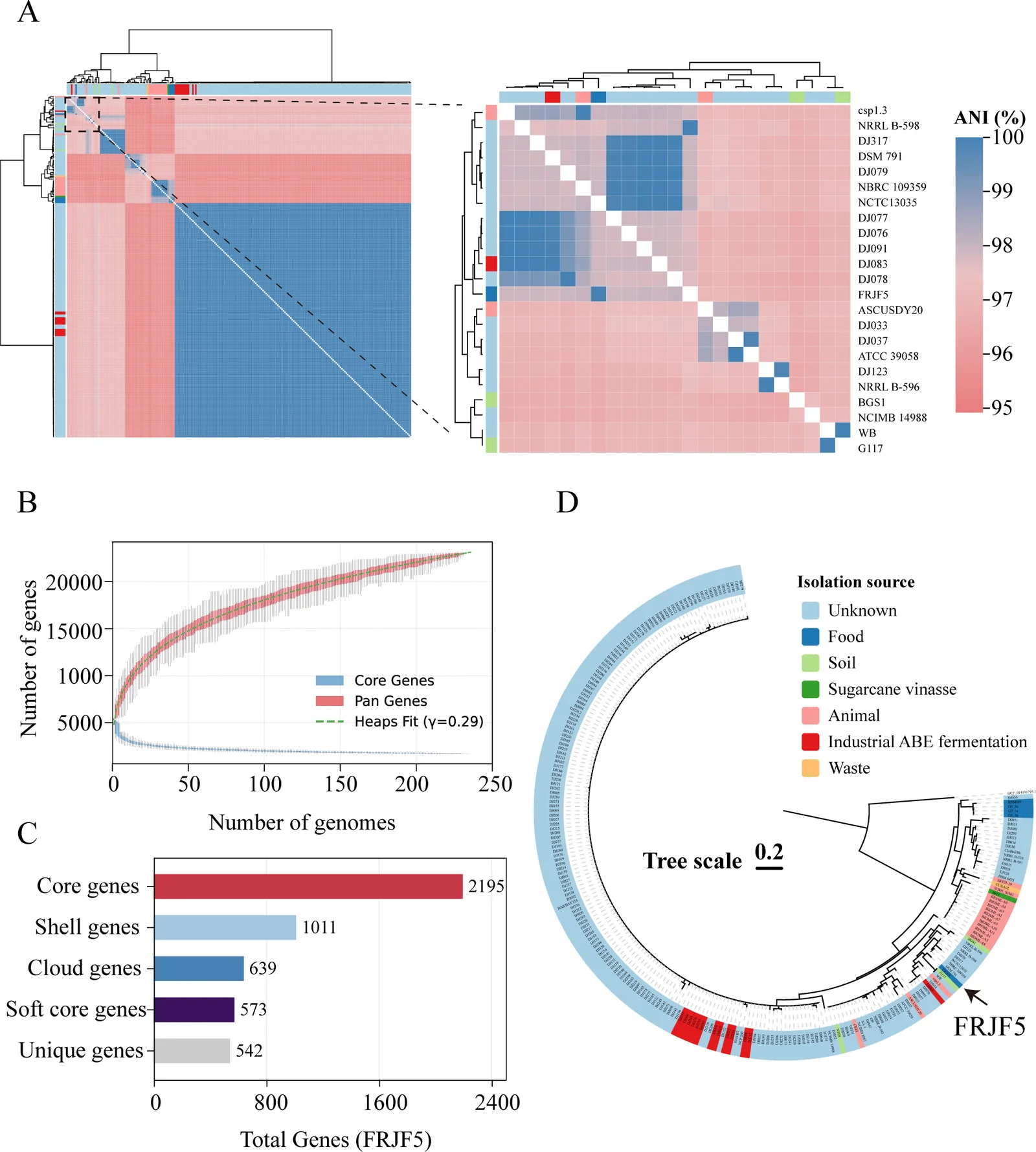
Comparison of *C. beijerinckii* FRJF5 with other 235 *C. beijerinckii* genomes. (**A**) A heat map of the ANI between all analyzed *C. beijerinckii* genomes. (**B**) Mathematical extrapolation for estimating the size of *C. beijerinckii* pan-genome and core genome. (**C**) The composition of the pan-genome of strain FRJF5. (**D**) Phylogenetic tree of *C. beijerinckii* genomes inferred from genomic data using the maximum likelihood method, with *C. butyricum* (GCF_014131795.1) as the outgroup.

A total of 235 *C. beijerinckii* genomes were retrieved and compared with FRJF5 (details are summarized in Data S1). All pairwise ANI values among these strains exceeded 95% (Fig. 3A). The highest ANI (98.01%) was observed between *C. beijerinckii* FRJF5 and *C. beijerinckii* csp1.3. Furthermore, to assess the diversity among *C. beijerinckii* strains, a pan-genome analysis was performed (Fig. 3B and C). We identified a closed pan-genome consisting of 23,086 gene clusters (Fig. 3B), which included a core gene set (9.76%, 2,253 genes), an accessory gene set (61.48%, 14,194 genes), and a strain-specific gene set (28.76%, 6,639 genes). The large accessory gene set reflects a high level of genetic diversity within this species. Projection analysis revealed a strong open trend in the pan-genome as more strains are added, indicating that further sampling would likely yield a considerable number of novel genes (Fig. 3B). The results of ANI clustering and genomic phylogenetic analysis were consistent (Fig. 3A and D). Notably, the strains clustering in the same branch as *C. beijerinckii* FRJF5 were isolated from diverse sources, including industrial ABE fermentation tanks, animal intestines, soil, and samples with unspecified origins.

#### Gene display of flavone-biotransforming genes and butyrate synthesis genes

**Fig 4:**
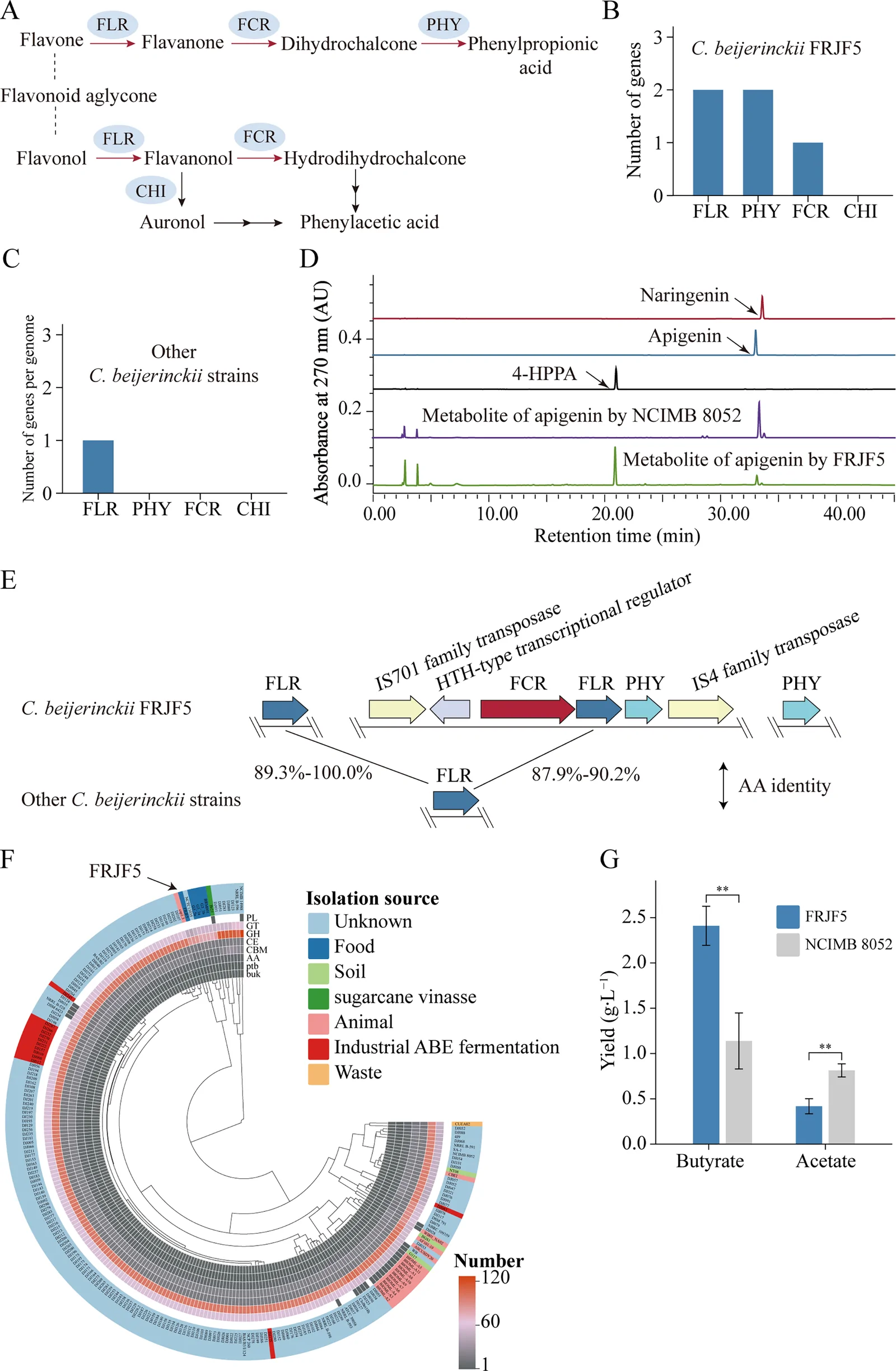
Comparative analysis of flavonoid transformation genes and butyrate-production-related genes across *C. beijerinckii* strains. (**A**) Bacterial biotransformation pathways of flavonoids. (**B**) Flavonoid biotransformation genes in strain FRJF5. (**C**) Flavonoid biotransformation genes in other *C. beijerinckii* strains (excluding FRJF5). (**D**) Apigenin transformation by *C. beijerinckii* NCIMB 8052 and strain FRJF5. (**E**) Overview of flavonoid biotransformation genes in *C. beijerinckii*. (**F**) Overview of carbohydrate-active enzymes and butyrate synthesis genes in *C. beijerinckii*. (**G**) Acetate and butyrate production by strains FRJF5 and NCIMB 8052 in RCM. Bars represent mean ± SD (*n* = 3). Statistical analysis was performed using an unpaired two-tailed Student’s *t*-test. **P < 0.01.

Flavonoids are a class of polyphenolic compounds that are widely found in plants and abundant in plant-based foods and known to provide various benefits to human health (38). Existing studies have shown that certain *Clostridium* species exhibit transformation activity toward plant flavonoids. To investigate whether *C. beijerinckii* strains, including *C. beijerinckii* FRJF5, possess flavonoid conversion capability, we surveyed the genomes of *C. beijerinckii* based on the known isoflavone conversion gene profile (Fig. 4A). The results revealed that only strain FRJF5 simultaneously possesses *flr*, *phy*, and *fcr* genes (Fig. 4B). In contrast, other *C. beijerinckii* strains generally only carry the *flr* gene (Fig. 4C). To verify whether these genes are functional, we selected the type strain *C. beijerinckii* NCIMB 8052 and strain FRJF5 for apigenin conversion experiments. The results confirmed that strain FRJF5 can convert apigenin into 3-(4-hydroxyphenyl)-propionic acid (4-HPPA), while strain NCIMB 8052 could only catalyze the first step, transforming apigenin into naringenin (Fig. 4D). This indicates that the predicted flavonoid conversion genes in the genome of *C. beijerinckii* FRJF5 are functional, and the prediction results are reliable. Furthermore, examination of gene arrangement revealed that *C. beijerinckii* FRJF5 harbors an *fcr-flr-phy* gene cluster flanked by transposases (Fig. 4E). This suggests that *C. beijerinckii* inherently possesses flavonoid reduction capability but lacks the ability to perform downstream reactions such as ring opening and hydrolysis.

On the other hand, *C. beijerinckii* is widely recognized for its butyrate-producing capability (39). We found that strain FRJF5 possesses the capacity for carbohydrate utilization, and the direct butyrate synthesis genes *ptb* and *buk* were detected in its genome (Fig. 4F). To further quantify its butyrate production, we measured the butyrate yields of strain FRJF5 and the type strain *C. beijerinckii* NCIMB 8052 using HPLC. The results showed that strain FRJF5 achieved a higher butyrate titer (2.41 g·L⁻¹) in RCM medium compared to *C. beijerinckii* NCIMB 8052 (1.14 g·L⁻¹; Fig. 4G).

#### Analysis of virulence factors and antibiotic resistance factors

**Fig 5:**
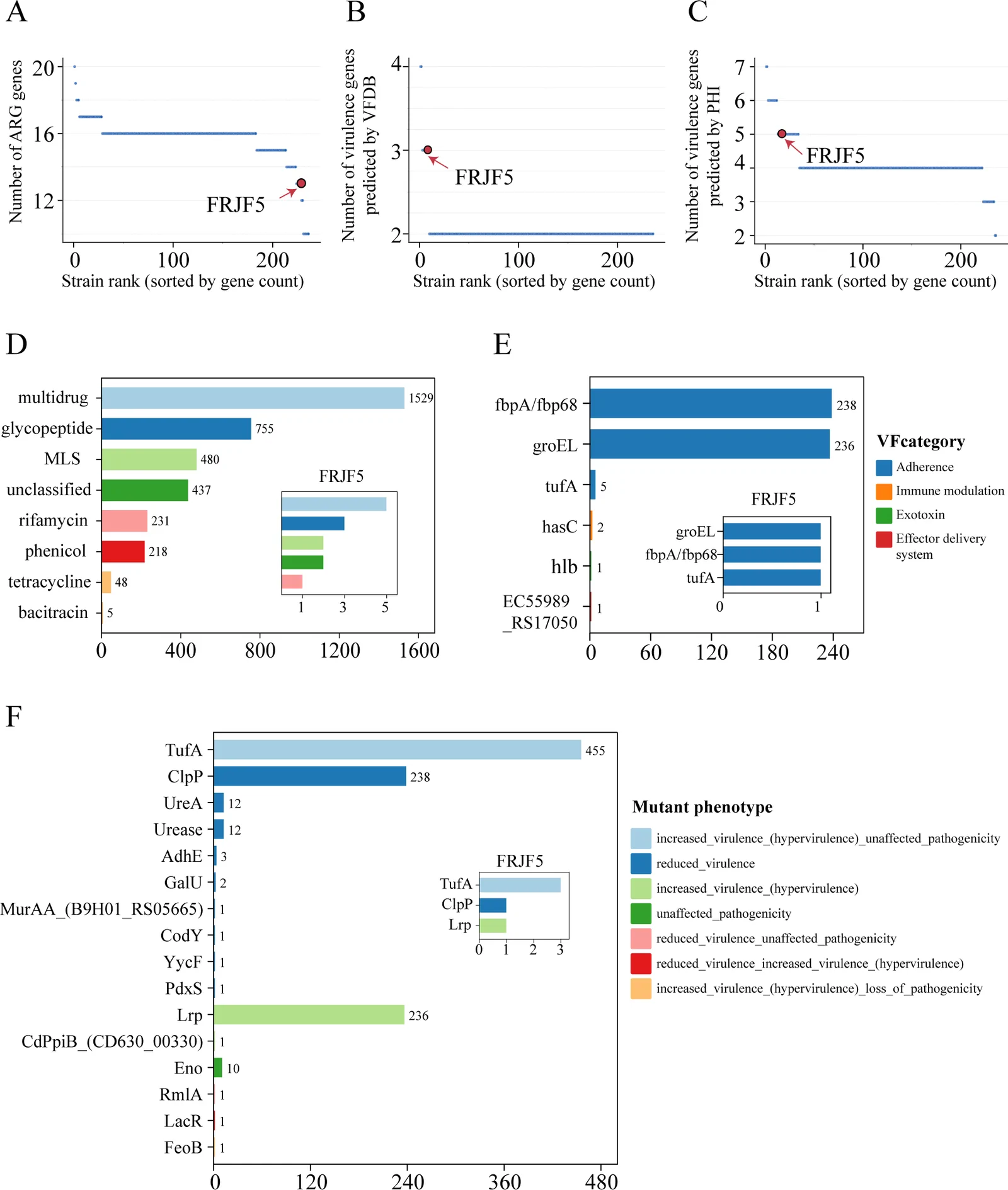
Representation of virulence genes/factors and ARGs identified in *C. beijerinckii* FRJF5 and *C. beijerinckii* genomes. Statistics of the number of antibiotic-resistance genes (**A**) and virulence factors from VFDB (**B**) and PHI (**C**) in each strain, and statistics of the types of resistance genes (**D**) and virulence factors from VFDB (**E**) and PHI (**F**) in total strains.

To understand the antibiotic resistance and potential pathogenicity of *C. beijerinckii* FRJF5, we compared its resistance factors and virulence factors with those of other *C. beijerinckii* strains. The results showed that strain FRJF5 does not harbor a large number of ARGs (Fig. 5A). Screening through the VFDB platform and PHI database revealed that the genome of strain FRJF5 contains a relatively large number of putative virulence-associated genes (Fig. 5B and C).

Analysis of ARGs in the genome of strain FRJF5 indicated the presence of genes conferring resistance to rifamycins, glycopeptides (*VanS*, *VanR*, and *VanRI*), and other unclassified antibiotics (Fig. 5D). It should be noted that no corresponding antibiotic resistance was observed in phenotypic antimicrobial susceptibility tests (Table 2) in strain FRJF5. Although a relatively large number of putative virulence-associated genes were predicted, most of these genes are species-intrinsic and are commonly present within the clade, including genes related to adhesion (Fig. 5E) as well as some virulence factors that are not inherently toxic. It should be noted that *Lrp*, a regulatory factor potentially involved in virulence, was not uniquely present in the genome of strain FRJF5 and was also commonly found in other *C. beijerinckii* strains (Fig. 5F). Interestingly, comparative analysis with other *C. beijerinckii* strains showed that strain FRJF5 possesses a higher copy number of elongation factor Tu-A (*tufA*) (Fig. 5E and F). Overall, no virulence factors with clearly documented toxicity were detected in strain FRJF5.

**TABLE 2:** Genome characteristics of *C. beijerinckii* FRJF5

Feature	Chromosome	Plasmid 1	Plasmid 2
Total length (bp)	5,728,266	70,925	7,793
GC content (%)	29.84	28.86	27.52
Topology	Circular	Not circular	Circular
tRNA	93	0	0
5s rRNA	16	0	0
16s rRNA	15	0	0
23s rRNA	15	0	0
sRNA	0	0	0
Gene number	5,261	78	12

**Fig 6:**
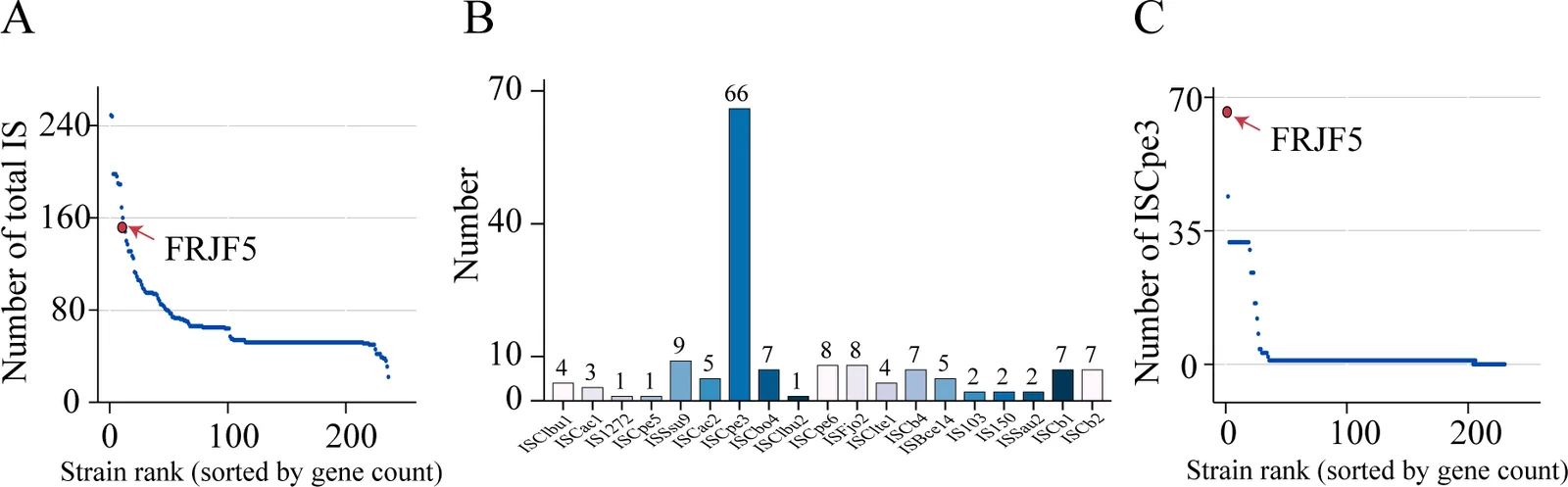
Overview of ISs identified in *C. beijerinckii* strains. (**A**) The frequency of total IS in the investigated *C. beijerinckii* strains. (**B**) The IS of FRJF5 strains and (**C**) the frequency of ISCpe3 in *C. beijerinckii* strains.

The major mobile genetic elements in *C. beijerinckii* strains were predicted and analyzed using ISfinder and BLASTn. A total of 149 ISs were detected in the genome of *C. beijerinckii* FRJF5. Among all 236 analyzed *C. beijerinckii* strains, the IS abundance of strain FRJF5 ranked in the top 10% (Fig. 6A). A total of 19 different IS types were identified in strain FRJF5 (Fig. 6B), most of which belong to the IS256 and IS3 families. The abundance of these IS elements reflects the genomic plasticity of FRJF5. It is noteworthy that, compared to other *C. beijerinckii* strains, ISCpe3 was the most abundant IS type in strain FRJF5 (*n*  =  66; Fig. 6C). ISCpe3 is a bacterial IS belonging to a widely distributed class of mobile genetic elements. The transposase it encodes catalyzes the movement of itself or adjacent genes within the genome, directly driving genomic recombination and variation. The high copy number of ISCpe3 suggests that strain FRJF5 may possess greater genomic plasticity and a potential adaptive advantage in responding to environmental changes and evolutionary pressures.

### *In vitro* safety assessment

#### The antibiotic resistance

Based on the previous comparative genomic analysis, the antibiotic resistance and potential pathogenicity of *C. beijerinckii* FRJF5 were predicted. Although no virulence factors with clearly defined toxicity were identified, potential resistance genes that may confer resistance to rifamycins, glycopeptides, and other unclassified antibiotics were predicted. To verify whether these genes actually confer antibiotic resistance to strain FRJF5, we conducted phenotypic antibiotic susceptibility testing. The results showed that strain FRJF5 was sensitive to a range of antibiotics, including ampicillin, tetracycline, clindamycin, cefoxitin sodium, doripenem, moxifloxacin, chloramphenicol, and metronidazole (Table 2).

#### Hemolytic activity

**Fig 7:**
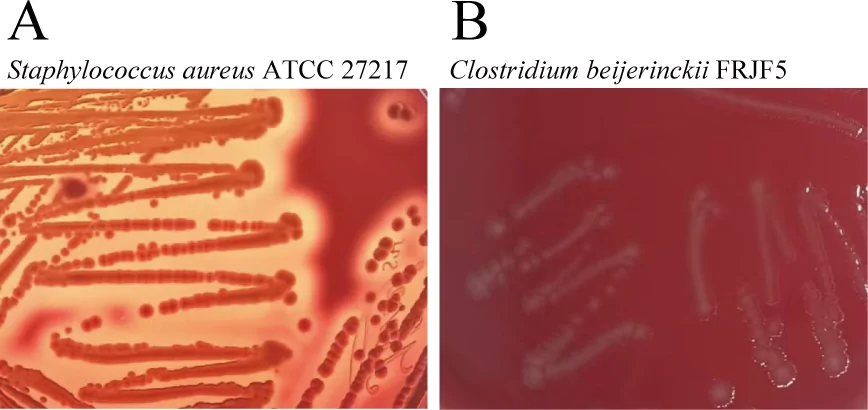
Growth of *S. aureus* ATCC 27217 (**A**) and *C. beijerinckii* FRJF5 (**B**) on the surface of blood agar after 24 h incubation at 37°C.

Hemolysis indicates that bacteria are capable of producing hemolysins, which can damage other cells. Many classic pathogenic bacteria exhibit hemolytic activity; therefore, hemolysis serves as an important marker of potential pathogenicity. We performed a hemolysis assay on *C. beijerinckii* FRJF5, using hemolysis-positive *S. aureus* as a positive control. The results showed a clear transparent zone of hemolysis around *S. aureus*, while no such zone was observed for strain FRJF5 (Fig. 7), indicating that strain FRJF5 does not possess hemolytic activity.

### *In vivo* safety evaluation

To further assess the in *vivo* safety of *C. beijerinckii* FRJF5, which was isolated from stinky tofu, we performed an oral gavage experiment on C57BL/6J mice using this strain. The results showed that the mice gavaged with strain FRJF5 exhibited good appetite, normal water intake and defecation, normal mental state, and normal activity levels. No abnormal behavior or mortality was observed in these mice over the 2-week gavage period. There was no significant difference in food intake (Table S1 in the supplemental material) or body weight (Table S2 in the supplemental material) between the mice administered the bacterial suspension and those in the control group (*p* > 0.05), which received an equivalent volume of physiological saline. At the conclusion of the experiment, the mice were anesthetized and euthanized for necropsy. Examination of the major organs, including the heart, liver, spleen, kidneys, and intestines, revealed no macroscopically visible pathological signs such as swelling, hemorrhage, or adhesions.

### Intervention trial in mice with colitis

#### Relevant indicator analysis

**Fig 8:**
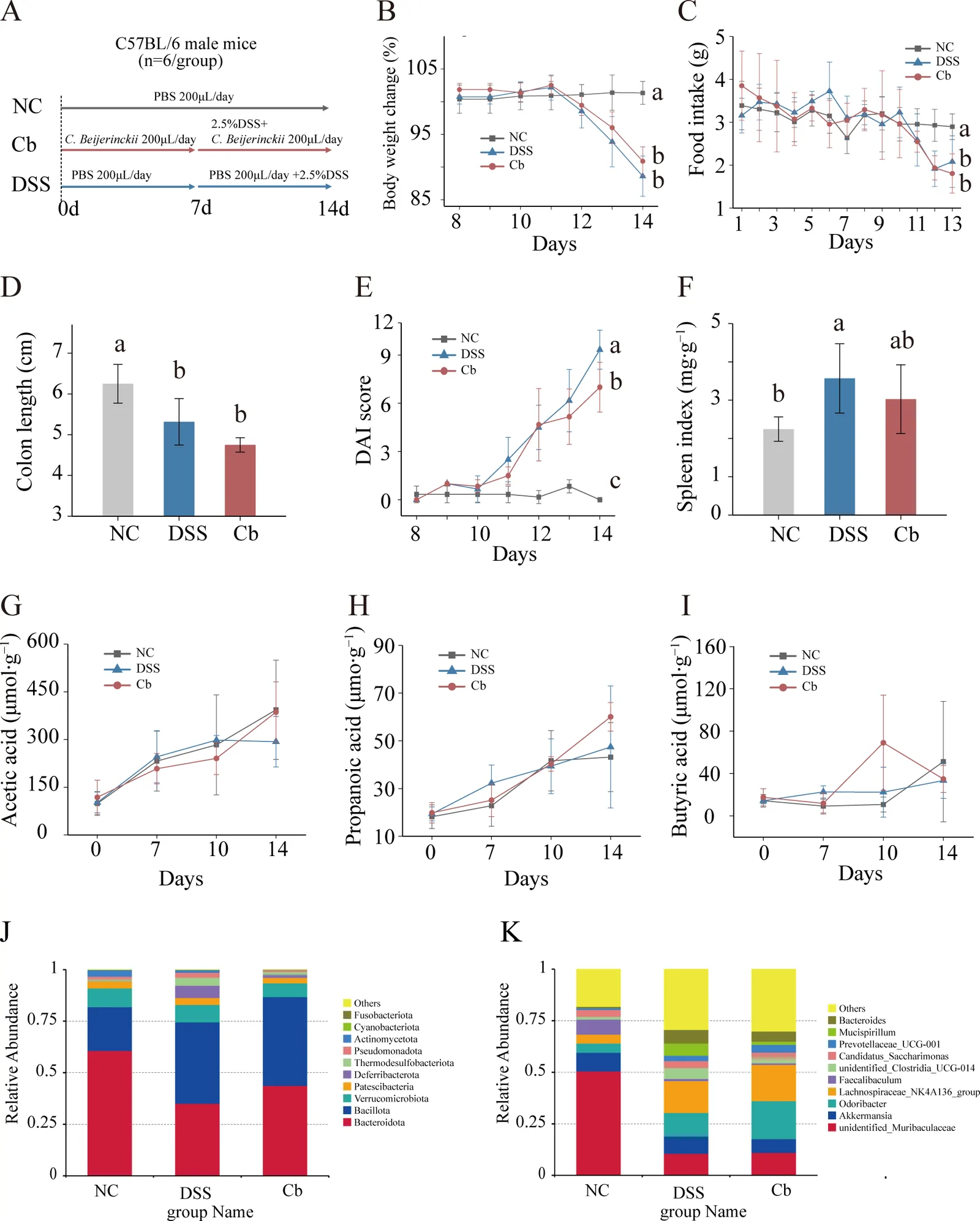
Effect of strain FRJF5 administration on mice colitis. (**A**) Schematic representation for DSS-induced colitis with administration of Cb as intervention. (**B**) Changes in body weights (**G**) of mice (*n* = 6). (**C**) Daily food intake of mice (*n* = 6). (**D**) Statistics of colon length. (**E**) Daily DAI scores (*n* = 6). (**F**) Spleen index of each treatment group (*n* = 6). Effects of different groups on the level of (**G**) acetic acid (*n* = 3), (**H**) propionic acid (*n* = 3), and (**I**) butyric acid in feces (*n* = 3). Comparison of the microbiota composition (**J**) at the phylum level of different groups and (**K**) at the genus level of different groups (*n* = 6). Data are presented as mean ± SD. Different letters indicate significant differences determined by one-way ANOVA followed by Waller-Duncan post hoc test (*P* < 0.05), while identical letters indicate no statistically significant differences (*P* ≥ 0.05).

The strictly anaerobic bacterium *C. beijerinckii* FRJF5 is not only capable of converting soy isoflavones into *O*-DMA via ring opening under anaerobic conditions but also produces butyrate during its growth. Research by Fan et al. (16) has demonstrated that butyrate-producing *C. beijerinckii* R8 isolated from lamb feces can modulate intestinal disorders in newborn lambs. To explore the potential applications of *C. beijerinckii* FRJF5, we conducted an intervention trial in a colitis mouse model (Fig. 8A), aiming to provide a theoretical and data-driven basis for expanding its utilization. The results are presented in Fig. 8. Compared to the NC group, the mice in the DSS group exhibited significant reductions in body weight (Fig. 8B), food intake (Fig. 8C), and colon length (Fig. 8D) (*P* < 0.05). No significant differences were observed between the Cb and DSS groups in terms of body weight, food intake, and colon length (*P* > 0.05). From days 8 to 14 (Fig. 8E), the DAI score in the DSS group increased sharply, while the increase in the Cb group was relatively gradual. By day 14, the DAI score of the Cb group was significantly lower than that of the DSS group (*P* < 0.05) but remained significantly higher than that of the NC group (*P* < 0.05). This result suggests that gavage with strain FRJF5 may have a moderate effect on alleviating colitis symptoms in mice. Compared to the NC group, the spleen index of the DSS group was significantly elevated (*P* < 0.05). Although the spleen index in the Cb group was lower than that in the DSS group, the difference was not statistically significant (Fig. 8F). Additionally, we analyzed SCFAs in mouse feces and found no significant differences in the levels of acetate (Fig. 8G), propionate (Fig. 8H), and butyrate (Fig. 8I) among the NC, DSS, and Cb groups. Notably, the butyrate concentration in the Cb group showed a rapid, linear increase from days 7 to 10, followed by a linear decline thereafter (Fig. 8I). The underlying reasons for this pattern warrant further investigation in future studies.

#### Gut microbiota analysis

To investigate whether gavage with *C. beijerinckii* FRJF5 can modulate the gut microbiota in colitis mice whose microbial community is known to be disrupted by DSS induction, we compared the gut microbial diversity among the NC, DSS, and Cb groups (Fig. 8J and K). At the phylum level, compared to the NC group, the DSS group exhibited a significant decrease in the relative abundance of Bacteroidota and a significant increase in Bacillota (*p* < 0.05). In the DSS group, the relative abundance of Bacteroidota was significantly reduced. Although a modest recovery was observed in the Cb group compared to the DSS group, no statistically significant differences were found between the Cb group and either the DSS or NC group (*P* > 0.05). Furthermore, compared to the NC group, the DSS group exhibited significantly higher relative abundances of Deferribacterota and Thermodesulfobacteriota (*P* < 0.05). In the Cb group, the relative abundance of Deferribacterota was lower than that in the DSS group, but no statistically significant differences were observed when compared to either the DSS or NC group (*P* > 0.05). These results indicate that gavage with FRJF5 has a certain restorative effect on the DSS-induced alterations in the abundances of Bacteroidota, Deferribacterota, and Thermodesulfobacteriota in colitis mice (Fig. 8J).

At the genus level, compared to the NC group, the DSS group showed a significant increase in the relative abundances of *Mucispirillum* and *Bacteroides* (*P* < 0.05). In the Cb group, these abundances were reduced relative to the DSS group, but no statistically significant differences were observed when compared to either the DSS or NC group (*P* > 0.05). These results suggest that gavage with FRJF5 exerts a modulatory (or restorative) effect on the DSS-induced elevation in the relative abundances of *Mucispirillum* and *Bacteroides* in mice with colitis (Fig. 8K).

## DISCUSSION

Soybeans are not only rich in high-quality protein and other essential nutrients, but they also contain functional components such as isoflavones, which have been associated with various health benefits (40). However, naturally occurring soy isoflavones primarily exist in glycosylated and methylated forms, which cannot be directly absorbed by the intestine. These compounds are metabolized by the intestinal microbiota into bioactive forms that become bioavailable. Thus, the gut microbiota plays a crucial role in the metabolism of ingested soy isoflavones (41, 42). Several specific intestinal bacteria capable of converting soy isoflavone daidzein into *O*-DMA have been identified from human and animal microbiota, including *Eubacterium ramulus* wK1 (43), *Clostridium* sp. HGHA136 (44), and *Clostridium* rRNA cluster XIVa strain SY8519 (45). Current research findings confirm that *O*-DMA, a microbial metabolite of soy isoflavone daidzein, exhibits stronger bioactivity than its parent compound, including enhanced estrogenic effects (46), superior antioxidant properties (12, 47), greater reduction of bone loss (48), and increased inhibition of cancer cell proliferation (49). In 2020, Peirotén et al. detected *O*-DMA in soymilk fermented with certain known lactic acid bacteria and reported that *Limosilactobacillus mucosae* INIA P508 yielded the highest concentration (21.32 ± 1.21 µmol·L^−1^) (50). Due to the low *O*-DMA product, in 2022, Landete successfully enhanced the concentrations of *O*-DMA and other bioactive compounds in soymilk by using genetically engineered lactic acid bacteria. Efforts are now underway to develop fermented soymilk rich in *O*-DMA and 6-OH-*O*-DMA into a functional food product (14). We detected *O*-DMA in the traditional Chinese fermented soybean product known as stinky tofu. While *O*-DMA has long been recognized as a metabolite of the soy isoflavone daidzein produced through intestinal microbial transformation, this study, to our knowledge, is the first to report the natural occurrence of *O*-DMA in a traditionally fermented soybean food. Through this research, we isolated a strictly anaerobic bacterium from Chinese stinky tofu that is capable of converting daidzein to *O*-DMA. This strain, FRJF5, was identified as *C. beijerinckii*.

Although previous studies have isolated *O*-DMA-producing bacteria from human fecal samples, none have reported the maximum concentration at which these strains could efficiently convert the substrate daidzein into *O*-DMA. In our earlier work, we isolated *O*-DMA-producing strains, *E. ramulus* Julong 601, *Enterococcus hirae* AUH-HM195, and *Clostridium* sp. AUH-JLC108, from human fecal samples (51), the feces of *Crossoptilon mantchuricum* (52), and chickens (21), respectively. All these strains were capable of efficiently converting daidzein to *O*-DMA in BHI medium at concentrations exceeding 300 µmol·L^−1^. In contrast, *C. beijerinckii* FRJF5 that had been isolated from stinky tofu in this study, efficiently converted daidzein in BHI medium at a concentration of 100 µmol·L^−1^, which is lower than the conversion capacities observed in the three animal-derived strains. We speculate that the source of isolation may influence the ability of the strain to transform soy isoflavones. Notably, phylogenetic analysis based on whole-genome sequences revealed that *C. beijerinckii* FRJF5 isolated from stinky tofu clustered most closely with *C. beijerinckii* strains from industrial ABE fermentation environments, soil, and animal feces, indicating their close genetic relationship. However, since the genes involved in *O*-DMA biosynthesis have not yet been characterized, it remains unclear whether other closely related *Clostridium* strains from different sources possess *O*-DMA-producing capability. It should be noted that *O*-DMA is a chiral compound. In this study, the e.e. of *O*-DMA produced by strain FRJF5 was 78.6% (Fig. 1D). Previous studies reported e.e. values of 90.0%, 66.9%, and 88.3% for *O*-DMA produced by *E. ramulus* Julong 601 (51), *Enterococcus hirae* AUH-HM195 (52), and *Clostridium* sp. AUH-JLC108 (21), respectively. To date, there have been no reports on differences in biological activity between *O*-DMA enantiomers. Further studies on the activity of different *O*-DMA enantiomers are necessary to provide a safety basis for the development of *O*-DMA as a pharmaceutical or health supplement.

To further evaluate the *in vivo* peobiotic potential of *C. beijerinckii* FRJF5, we established a colitis mouse model and administered *C. beijerinckii* FRJF5 via gavage. Surprisingly, only the DAI score indicated a significant effect of FRJF5 gavage on maintaining intestinal health in colitis mice (Fig. 8E). The spleen index of the DSS group was significantly higher than that of the NC group. Although the spleen index of the Cb group showed a decreasing trend compared to the DSS group, the difference was not statistically significant. However, it should be noted that the spleen index of the Cb group also did not differ significantly from that of the NC group. Therefore, we speculate that gavage with strain FRJF5 may help control the progression of splenic inflammation in colitis mice. Our results show that at the genus level, the relative abundance of *Mucispirillum* was significantly higher in the DSS group compared to the NC group (*P* < 0.05). Compared to the DSS group, the Cb group showed a notable decrease in the relative abundance of *Mucispirillum*, though the difference was not statistically significant. It is worth noting that the relative abundance of *Mucispirillum* in the Cb group also did not differ significantly from that in the NC group. These results suggest that gavage with strain FRJF5 may partially restore the abundance of *Mucispirillum* in colitis mice. It is possible that with prolonged gavage duration, strain FRJF5 could significantly suppress the DSS-induced increase in the relative abundance of *Mucispirillum*. The genus *Mucispirillum* colonizes the intestinal mucus layer, and its effects depend on the host’s immune status. In 2019, Caruso et al. found that in genetically modified mice (Nod2^−/−^Cybb^−/−^), the genus *Mucispirillum* accumulated in the intestine and triggered colitis resembling Crohn’s disease (53). Taken together, our study identified *C. beijerinckii* FRJF5 as a promising candidate strain for the simultaneous production of *O*-DMA and butyrate, highlighting its potential for future functional-food applications.

## Data Availability

The complete genome sequence of Clostridium beijerinckii FRJF5 has been deposited in GenBank under accession number JCAMPI000000000 (BioProject: PRJNA1481890). The 16S rRNA gene sequence of strain FRJF5 is available under GenBank accession number PX765463. The strain has been deposited in the China General Microbiological Culture Collection Center (CGMCC) under deposit number CGMCC 7.759. All other relevant data are available within the manuscript and its supplemental materials. Raw data and strains are available from the corresponding authors upon reasonable request.

## SUPPLEMENTAL MATERIAL

Data S1 spectrum.00632-26-s0001.xlsx *Clostridium beijerinckii* genomes information.

Tables S1 and S2 spectrum.00632-26-s0002.docx Effect of *Clostridium beijerinckii* FRJF5 on mice.

ASM does not own the copyrights to Supplemental Material that may be linked to, or accessed through, an article. The authors have granted ASM a non-exclusive, world-wide license to publish the Supplemental Material files. Please contact the corresponding author directly for reuse.
