# Supplementary material for: Isolation, genomic characterization, and safety assessment of an O-desmethylangolensin-producing Clostridium beijerinckii strain from Chinese Stinky Tofu
Source: Microbiol Spectr. 2026 Sep 17;14(10):e00632-26. doi: 10.1128/spectrum.00632-26 (PMC13689313; doi:10.1128/spectrum.00632-26)
Supplement: Supplementary file 1 [file spectrum.00632-26-s0002.docx]

Table S1. Effect of *Clostridium beijerinckii* FRJF5 on mice food intake.

| Gender | Group | food intake (g/d ) | | |
| --- | --- | --- | --- | --- |
|  |  | 0d | 7d | 14d |
| Male | control | 3.31 ± 0.36 | 3.00 ± 0.14 | 3.05 ± 0.51 |
|  | gavage FRJF5 | 3.20 ± 0.40 | 3.13 ± 0.62 | 3.06 ± 0.17 |
| Female | control | 2.10 ± 0.17 | 2.45 ± 0.17 | 2.59 ± 0.15 |
|  | gavage FRJF5 | 2.16 ± 0.17 | 2.48 ± 0.17 | 2.57 ± 0.20 |

**Footnotes:** Data are presented as mean ± SD (n = 6). Different lowercase letters within the same column indicate significant differences between groups as determined by one-way ANOVA followed by Waller-Duncan post hoc test (*p* < 0.05).

Table S2. Effect of *Clostridium beijerinckii* FRJF5 on mice body weight.

| Gender | Group | weight (g) | | |
| --- | --- | --- | --- | --- |
|  |  | 0d | 7d | 14d |
| Male | control | 22.88 ± 0.36 | 23.94 ± 0.74 | 24.24 ± 0.86 |
|  | gavage FRJF5 | 23.27 ± 0.36 | 23.59 ± 0.62 | 23.93 ± 0.65 |
| Female | control | 19.99 ± 0.64 | 20.76 ± 0.21 | 21.38 ± 0.29 |
|  | gavage FRJF5 | 20.18 ± 0.41 | 20.68 ± 0.33 | 21.57 ± 0.33 |

**Footnotes:** Data are presented as mean ± SD (n = 6). Different lowercase letters within the same column indicate significant differences between groups as determined by one-way ANOVA followed by Waller-Duncan post hoc test (*p* < 0.05).
